# Mr. Chamberlaine, on Opinions in Courts of Justice

**Published:** 1802-03

**Authors:** W. Chamberlaine

**Affiliations:** Aylesbury Street, Clerkenwell


### To Dr. BRADLEY.

"Life and Death are objects too important to be fported with in the "manner they fometimes are; nor fhould the valuable connexions of "our Fellow-citizens be ever facrificed to the ignorance of the Faculty, "the caprice of a Court, or the artifices of revenge and difappoint-"ment."

Dear Sir,

A Circumftance which has lately occurred, points out the juftice of the above obfervation, which I have quoted from the concluding fentence of the Preface of a very excellent little Work, which, I think, ought to be in the hands of every Medical Practitioner and every Coroner;* and fhews how very cautious medical men fhould be in giving their opinion in cafes where life and death may depend on their decifion.

On the 29th of December, I examined, in company with Mr. Bartlett, Apothecary of the Finfbury Difpenfary, the body of a new-born infant which had been found in a privy. The child was come to its full time, and it was very evident that the mother had had a very tedious and laborious parturition. All the vifcera were in a perfectly found ftate. On examining the lungs, I found them of a *bright red* colour, and they did not completely fill up the cavity of the thorax. On throwing a portion into water, it rofe to the furface; this circumftance, however, had no *weight* with me, as it is well known that many caufes may operate to render the lungs of a *still-born* child fpecifically lighter than water. But the appearance that principally guided me in my opinion on the fubject, delivered on oath before the Coroner, was, that of the infant having its hands *very strongly clinched*; I have been feveral years in the practice of Midwifery, and during that period have delivered many ftill-born children, but never one in which that circumftance had taken

* Farr's Medical Jurifprudence, fmall 8vo. pp. 140. Becket, London, 1788.

taken place; nor could I, by any mode of reafoning that I could adopt, reconcile it to my ideas how that could happen, unlefs induced by fpafm, (except in cafe of mal-formation, which was not the cafe here,) and that fpafm could not be brought on, independent of the exiftence of life. In this opinion I was afterwards in fome meafure ftrengthened, by the authority of Dr. Farr, who, in the work I have already quoted, obferves, (p. 65,) "We fhould obferve likewife, whether it has grafped any thing in its hands, *as this is a certain proof of life.*"

Under this impreffion, I delivered my opinion to the fame effect, when examined before the *Grand Jury.*

Between this period, and the impending trial of the mother of the child, at the Old Bailey, I determined to have the beft information that books, and confultations with profeffional gentlemen, whofe anatomical refearches and enquiries had given them far greater knowledge of the fubject than any opportunities of my own could afford me; I reflected, that though it might have been born with life, yet the circumftance of the funis umbilicus being, by the violence of its fall, broke off about four inches from the abdomen, might have occafioned a mortal hæmorrhage;* although this could not be afcertained, as the body of the infant had been wafhed clean. I alfo confidered, that it might have been ftunned by receiving a blow in its fall down the privy, which the appearance of a contufion on the fide of its head rendered probable; and I had my doubts, relative to the completion of the procefs of circulation and infpiration, in confequence of the appearance of the colour of the lungs. I have never had an opportunity of examining the vifcera of a child actually known to be ftill-born; but well knowing that where breathing and circulation have been perfected, the lungs are of a livid whitifh colour, and fill up the cavity of the thorax, I ftated my doubts to fome gentlemen; and among others, to Mr. Blair, † who not only confirmed my opinion as to the poffibility that perfect refpiration might not have taken place, but ftated a circumftance which plainly demonftrated the ground on which I *principally* refted my opinion of the child having been born alive, to be erroneous. He affured me, that not

* Hæmorrhage does not always take place when the funis is broken off by violence. A lady, one of my beft patients, was taken fo fuddenly in labour, that the child, if I may ufe the expreffion, *shot forth* from her with fuch force as to feparate the funis, which broke exactly in the right place, and as *even* as if it had been cut with fciffars; not fo much as one drop of blood followed, although the child was ftrong, and very lively.

† As Mr. Blair permitted me to mention his name in the Court, as my authority for changing my firft opinion, I flatter myfelf he will not be offended at my again taking the fame liberty in this place.

not long ago, a labour had occurred to him, in which the child, which was ftill-born, and had been dead four or five days before delivery, had *both* its *hands* VERY FIRMLY CLINCHED.

It is no difcredit to any man to retract an erroneous opinion; obftinately to perfift in it, againft conviction, where both the life of a fellow-creature and my own reputation were at ftake, would be downright murder. In giving my teftimony on the day of trial, at the Old Bailey, I ftated the circumftance related to me by Mr. Blair, (who humanely gave his attendance in the Court) as my reafon for changing my opinion. Mr. Blair's eminence in his profeffion, and high refpectability, had the fame weight with the Court and Jury as with me, for the Jury were perfectly fatisfied without requiring that gentleman's depofition on oath; and as the fole queftion then lay in a fmall compafs, namely, whether the child was born dead or alive; and as this latter could not be proved, but rather the reverfe, the prifoner was of courfe acquitted.

I have thought it not altogether improper to trouble you with this Cafe, on reflecting how many lives may have been facrificed by too hafty an opinion given, or a decifion made, on grounds erroneous, or not fully confidered! How many executions have taken place, of mothers for the murder of their infants, from the fingle circumftance of the *lungs swimming in water!* which no enlightened practitioner will now place any reliance on; but which has been, and may in future be, the caufe of much mifchief in places remote from the capital, where opportunities of knowing better have not been afforded.

I am, &c.

W. CHAMBERLAINE.

*Aylesbury Street, Clerkenwell, Feb.* 21, 1802.

